# Neonatal familial cold autoinflammatory syndrome: A newborn with recurrent cold-induced urticarial eruptions

**DOI:** 10.1016/j.jdcr.2025.10.053

**Published:** 2025-11-04

**Authors:** Tomassoni Nicholas, Allen Mary Grace, Toal Kaitlin, Shenberger Keith, Cho Youngjin

**Affiliations:** a Geisinger College of Health Sciences School of Medicine, Scranton, Pennsylvania; b Department of Rheumatology, UPMC Williamsport, Williamsport, Pennsylvania

**Keywords:** CAPS, Cold-induced urticaria, Cryopyrin-associated periodic syndromes, Familial cold autoinflammatory syndrome, FCAS, IL-1 blockade, Infant, NLRP3 mutation, Periodic fever and rash

## Introduction

Familial cold autoinflammatory syndrome (FCAS) is a rare autoinflammatory disease occurring in approximately 1 in every 1-2 million inhabitants in the United States.^1^ FCAS belongs to the cryopyrin-associated periodic syndromes (CAPS), a spectrum of 3 related conditions that include Muckle-Wells syndrome (MWS) and neonatal-onset multisystem inflammatory disease (NOMID).^2^, ^3^, ^4^ The distinctive cold-induced urticaria is one of the most recognizable dermatologic features for early identification of FCAS in infants, particularly where other symptoms may be difficult to assess. Early recognition of FCAS is critical for initiating IL-1 targeted therapy, which has dramatically improved patient outcomes.^5^ We present a case of severe neonatal FCAS in a patient with the L355P mutation, occurring within 5 consecutive generations. This case demonstrates the need for immediate intervention in severe systemic presentations and contributes valuable data as one of the earliest documented FCAS presentations with severe respiratory compromise.

## Case presentation

**Fig 1:**
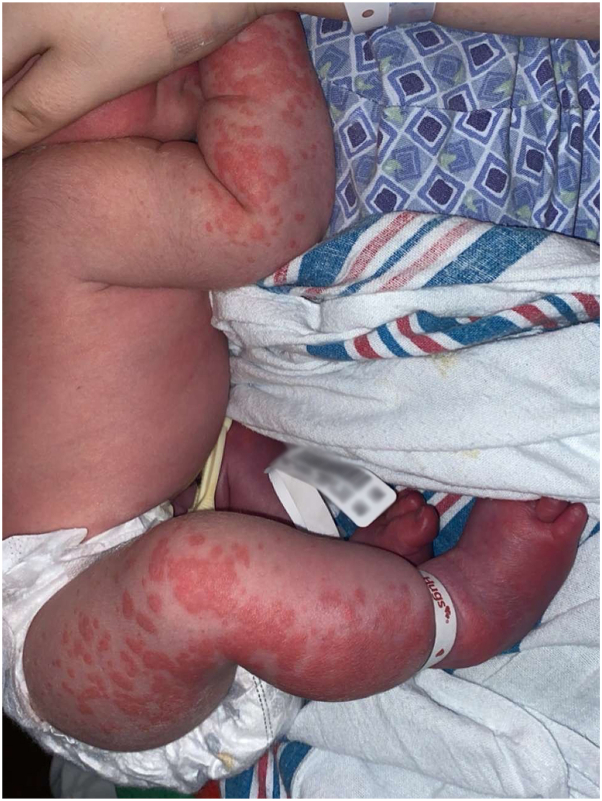
Cold-induced eruption in neonatal familial cold autoinflammatory syndrome at 14 hours of life. Clinical photograph demonstrates widespread blanching, erythematous patches and plaques on the extremities following routine delivery room cold exposure, typical of cold-induced inflammatory response in patients with FCAS, with purpuric areas visible on the lower legs. The eruption subsided after warming measures in the NICU.

Following routine delivery room cold exposure, a 39-week term newborn developed diffuse blanching, evanescent, erythematous patches, and confluent plaques on the extremities, with prominent involvement of the lower legs, where areas of purpura were also visible. The eruption subsided after warming measures. Cyanosis and oxygen desaturations presented immediately within days of birth, requiring neonatal intensive care unit (NICU) intervention (Fig 1).

**Fig 2:**
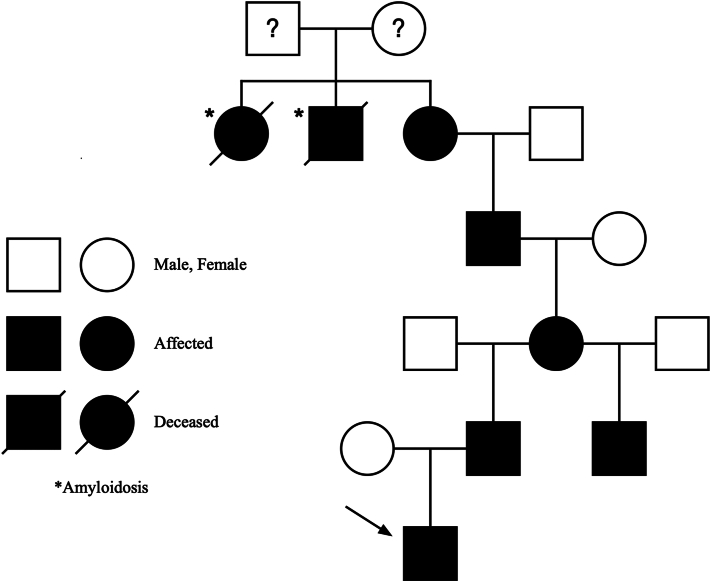
Five-generation pedigree of familial cold autoinflammatory syndrome inheritance pattern as reported by the patient’s family. A five-generation pedigree demonstrates an autosomal dominant inheritance pattern of FCAS within the patient's family. *Squares* represent males, *circles* represent females. Filled symbols indicate affected individuals with confirmed or clinically suspected FCAS. The *diagonal line* through symbols indicates deceased family members. The asterisk (∗) denotes a family member with documented amyloidosis. The *arrow* indicates the proband (patient described in this case report). Question marks indicate family members with unknown genetic status.

Vital signs were notable for the absence of fever and a normal heart rate. Laboratory evaluation was notable for elevated C-reactive protein (CRP, 3.5, reference < 1). Radiographic imaging revealed slight lung hypoinflation with no pathology. The family reported 5 generations diagnosed with FCAS (Fig 2) and a known NLRP3 (previously known as CIAS1) mutation.^6^

NICU admission and rheumatology referral were arranged. With CRP elevated to 12 mg/L, and based on clinical presentation and family history, off-label anakinra (an IL-1α and IL-1β antagonist) treatment (2 mg/kg, subcutaneous) was initiated at 11 days of life, pending genetic testing results. Given the overlapping phenotypes between FCAS and MWS and the risk of progression to MWS-like complications if untreated, screening for sensorineural hearing loss, inflammatory eye disease, and hepatosplenomegaly was performed. The negative findings, combined with clear cold-triggered urticarial presentations and family history, supported the FCAS diagnosis over MWS. The primary management included monitoring for oxygen desaturations and urticarial exacerbations. Upon discharge, the patient experienced multiple cold-induced urticarial exacerbations. The genetic testing confirmed a monoallelic NLRP3 mutation (c.1064T>C, L355P; alternatively, L353P), a known pathogenic variant for FCAS.^6^^,^^7^ In subsequent rheumatology follow-up, the medication dosage increased to approximately 3.5 mg/kg daily to manage breakthrough symptoms, including urticarial eruptions with fever. Following dose optimization, the patient had no subsequent cold-induced symptoms, showing excellent control with anakinra.

## Discussion

While FCAS and MWS present with overlapping phenotypes, the identification of cold-triggered inflammatory flares is typical of FCAS and serves as a key differentiating feature from MWS.^2^^,^^3^ Without prompt recognition and treatment, both conditions may progress to serious complications, including sensorineural deafness and amyloidosis.^2^^,^^3^

The clinical manifestations of FCAS result from specific genetic defects that disrupt normal inflammation. FCAS and all CAPS conditions result from mutations in the NLRP3 (NOD-like receptor family pyrin domain containing 3) gene, which encodes NLRP3/cryopyrin, a component of the inflammasome complex that regulates IL-1β production.^7^^,^^8^ While the detailed molecular mechanisms of pathogenic variants may vary, these mutations lead to excessive IL-1β release, causing symptoms of episodic fever, urticaria, arthralgia, and fatigue, which are typically triggered within hours of cold exposure and are mostly self-limiting. The L355P (L353P) variant was identified in large, multi-generational families with extensive pedigrees, accounting for the majority of FCAS cases reported in North America.^9^ FCAS episodes typically occur within 3 hours of cold exposure and resolve spontaneously within 24 hours. In contrast, our patient showed persistent, severe systemic symptoms from birth. The presence of cyanosis and oxygen desaturations non-responsive to supplemental oxygen extends beyond the well-known clinical spectrum of FCAS, which typically induces rash, fever, arthralgia, and conjunctivitis.

The successful use of anakinra in an 11-day-old neonate extends beyond current FDA approval guidelines: FDA approves neonatal use of anakinra in NOMID. Off-label use in neonates includes Kawasaki disease or hemophagocytic lymphohistiocytosis.^10^ There was consideration for a transition to canakinumab in the future, but anakinra is readily available to the patient. This case demonstrates successful treatment outcomes with anakinra in one of the youngest FCAS patients when appropriate dosing is achieved, supporting published evidence that anakinra prevents cold-induced FCAS episodes, providing valuable evidence for neonatal management.

This case emphasizes the importance of including FCAS in the differential diagnosis for severe, cold-induced neonatal urticaria, particularly when systemic symptoms are present. The combination of respiratory compromise, persistent urticaria, and family history distinguishes this case from benign rashes and highlights the need for careful evaluation of cold-triggered dermatologic manifestations.

Despite the extensive family history of FCAS, the clinical implication was not effectively understood by the early care team, resulting in diagnostic delay. This highlights how healthcare communication gaps can occur even with informed families when genetic information is not readily accessible at the point of care. Amyloidosis is very rare in patients with FCAS.^2^ With a familial report of a history of amyloidosis, this raises the question of whether delayed recognition and treatment of FCAS can contribute to the progression of secondary amyloidosis.

Early IL-1 blockades with anakinra or canakinumab can successfully control symptoms and may prevent long-term complications, such as amyloidosis and hearing loss. This case highlights the importance of improved communication systems between families and health care providers in ensuring the timely recognition and treatment of rare genetic conditions.

## Conflicts of interest

None disclosed.
